# Investigating the Association between Motor Function, Neuroinflammation, and Recording Metrics in the Performance of Intracortical Microelectrode Implanted in Motor Cortex

**DOI:** 10.3390/mi11090838

**Published:** 2020-09-03

**Authors:** Evon S. Ereifej, Youjun Li, Monika Goss-Varley, Youjoung Kim, Seth M. Meade, Keying Chen, Jacob Rayyan, He Feng, Keith Dona, Justin McMahon, Dawn Taylor, Jeffrey R. Capadona, Jiayang Sun

**Affiliations:** 1 Departments of Neurology and Biomedical Engineering, University of Michigan, Ann Arbor, MI 48109, USA; 2 Veteran Affairs Ann Arbor Healthcare System, Ann Arbor, MI 48105, USA; 3 Advanced Platform Technology Center, Rehabilitation Research and Development, Louis Stokes Cleveland VA Medical Center, Cleveland, OH 44106, USA; mag150@case.edu (M.G.-V.); yjk14@case.edu (Y.K.); smm214@case.edu (S.M.M.); kxc399@case.edu (K.C.); jxr429@case.edu (J.R.); hxf89@case.edu (H.F.); krd37@case.edu (K.D.); jam399@case.edu (J.M.); dawn.taylor@case.edu (D.T.); jrc35@case.edu (J.R.C.); 4 Department of Population and Quantitative Health Sciences, Case Western Reserve University, Cleveland, OH 44106, USA; yxl1469@case.edu; 5 Department Biomedical Engineering, Case Western Reserve University, Cleveland, OH 44106, USA; 6 Department of Neurosciences, Cleveland Clinic Lerner Research Institute, Cleveland, OH 44106, USA; 7 Cleveland Functional Electrical Stimulation Center, Louis Stokes Cleveland Veterans Affairs Medical Center, Rehabilitation Research and Development, Cleveland, OH 44106, USA; 8 Department of Statistics, George Mason University, Fairfax, VA 22032, USA; 9 United States Department of Agriculture (USDA) Agricultural Research Service, Office of National Programs, Beltsville, MD 20705, USA

**Keywords:** intracortical microelectrode performance, motor behavior, inflammation, numerical formal concept analysis (nFCA), regression analysis

## Abstract

Long-term reliability of intracortical microelectrodes remains a challenge for increased acceptance and deployment. There are conflicting reports comparing measurements associated with recording quality with postmortem histology, in attempts to better understand failure of intracortical microelectrodes (IMEs). Our group has recently introduced the assessment of motor behavior tasks as another metric to evaluate the effects of IME implantation. We hypothesized that adding the third dimension to our analysis, functional behavior testing, could provide substantial insight on the health of the tissue, success of surgery/implantation, and the long-term performance of the implanted device. Here we present our novel analysis scheme including: (1) the use of numerical formal concept analysis (nFCA) and (2) a regression analysis utilizing modern model/variable selection. The analyses found complimentary relationships between the variables. The histological variables for glial cell activation had associations between each other, as well as the neuronal density around the electrode interface. The neuronal density had associations to the electrophysiological recordings and some of the motor behavior metrics analyzed. The novel analyses presented herein describe a valuable tool that can be utilized to assess and understand relationships between diverse variables being investigated. These models can be applied to a wide range of ongoing investigations utilizing various devices and therapeutics.

## 1. Introduction

Recordings from individual or small populations of neurons via intracortical microelectrodes (IMEs) has allowed patients with neurological disease and spinal cord injuries to use their thoughts to control computer cursors [1], robotic arms [2], as well as the patient’s own limb [3]. Despite the numerous successes utilizing IME technologies, widespread clinical implantation is currently impeded by difficulties in maintaining stable long-term recordings [4,5]. Inconsistencies in recording performance and overall decreases in recording quality has been observed in numerous animal models, including human, across several labs [6,7,8,9,10,11,12]. 

A major hurdle to the clinical implementation of IME remains to be the electrode instability caused in large part by the chronic inflammatory response [13]. The implantation of the IME severs vasculature, thus breaching the blood–brain barrier and initiating the inflammatory response [14,15,16]. Local microglia and infiltrating macrophages become activated, releasing cytokines, neurotoxic factors, and reactive oxygen species (ROS) [17,18,19,20,21]. The release of cytokines, neurotoxic factors, and ROS around implanted electrodes results in a perpetual foreign body response, death of neurons, and corrosion and delamination of the microelectrode surface [20,22,23,24,25,26,27,28]. In addition, astrocytes become reactive and build a high impedance glial scar around the electrode, barricading it from the healthy parenchyma [29]. Consequently, neurons are affected by the inflammatory response, either by the physical increased distance to the electrode or their resultant degeneration and death [19,30,31]. It is unclear if the decrease in electrode performance is due to the neuroinflammatory response resulting in the decline of neuron viability, degradation of the device materials, or both [32,33].

Unfortunately, few studies have found an association between the relationship of the microelectrode performance and the neuroinflammatory response of the implanted devices. Notably, some studies utilizing therapeutics have indicated that proinflammatory molecules and blood–brain barrier permeability have connections to recording performance and histological outcomes [34,35,36,37]. More recently, we have demonstrated that implanting microelectrodes in the motor cortex causes immediate and lasting motor deficits. Motor deficits were depicted by a significant increase in time required to complete a motor behavior task postimplantation [38]. Measured motor deficits were correlated to the chronic damage to the blood–brain barrier [38]. Our initial assessment of the motor behavior tasks did not include evaluation of the electrophysiological recordings, thus analysis of the correlation with recording performance was not performed.

The objective of this pilot study was to utilize a novel, network, numerical formal concept analysis (nFCA) and traditional correlation regression analyses to evaluate the relationships between electrophysiology, histology, and motor performance metrics. Here, a group of Sprague Dawley rats were implanted with intracortical microelectrodes for eight weeks. Throughout the duration of the experiment, we performed biweekly electrophysiological recordings as well as fine and gross motor behavior tests. Histological assessment for neuron density, blood–brain barrier permeability, activated microglia/macrophages, and reactive astrocytes was performed postmortem. Our comprehensive statistical analyses of the data have provided promising, interesting associations, which sets a basis for future studies. 

## 2. Materials and Methods 

### 2.1. Neural Probe Implantation Procedure 

All animal procedures were performed as approved by the Institutional Animal Care and Use Committee (IACUC) at the Louis Stokes Cleveland Department of Veterans Affairs Medical Center and all experiments were performed in accordance with relevant guidelines and regulations. Prior to implantation, impedance magnitudes were verified to the manufacturer’s values by measuring electrode impedances at 1 kHz in saline. All electrodes were cleaned with 95% ethanol and deionized water wash for five minutes each. Subsequently, implants were sterilized using the standard ethylene oxide (EO) gas protocol in our lab: 54.4 °F, 1hour sterile time and 12 h aerate [39]. 

Surgical procedures were similar to our previously published methods [12,20,40,41]. Six male Sprague Dawley rats (8–10 weeks old, ~225 gm) were implanted with silicon, single shank, 16 channel intracortical microelectrodes (NeuroNexus A1x16-3mm-100-177-Z16, NeuroNexus, Ann Arbor, MI, USA) in the primary motor cortex (2mm lateral to midline and 2mm anterior to bregma) for eight weeks. Rats were first anesthetized in an isoflurane chamber (3.5% in 1.5 L/min O_2_). The animals’ head were shaved around the incision site and sterilized with alternating chlorhexidine gluconate (CHG) and isopropanol scrubs. Marcaine (MWI Veterinary Supple, Boise, ID, USA) was administered subcutaneously (SQ) around the surgical site. Next, Carprofen (5 mg/kg) and Cefazolin (25 mg/kg) (MWI Veterinary Supple, Boise, ID, USA) were administered SQ for analgesic and antibiotics, respectively. Animals were then mounted on a stereotaxic frame with 0.5–2% isoflurane flowing through the nosecone for maintenance of anesthesia. A circulating water pad was placed under the animal to maintain body temperature at 37 °C. Animal body temperature and vitals were monitored using a MouseSTAT^®^ Pulse Oximeter and Heart Rate Monitor (Kent Scientific Corp., Torrington, CT, USA). 

Once the animal was fully prepared for surgery, an incision down the midline of the scalp was made, and the skin was retracted to view the skull. The skull was cleaned of the periosteum, dehydrated using hydrogen peroxide, and primed using Vetbond (3M, Maplewood, MN, USA) animal tissue adhesive. Craniotomies using a 0.45 mm drill bit were made for ground wire (1.5 mm lateral to midline and 1.5 mm posterior to bregma) and reference wire (1.5 mm lateral to midline and 5.5 mm posterior to bregma). The wires were manually inserted into their respective holes using fine forceps and secured onto the skull with Teets cold cure dental cement (AM Systems, Sequim, WA, USA). Following which, a 2 mm hole was drilled into the skull on the contralateral side, 2 mm lateral to midline and 2 mm anterior to bregma (primary motor cortex). The dura was reflected using an angled Von Graefe knife, and the implant was inserted using a micromanipulator. The micromanipulator was gradually lowered 100 microns (the distance between channels) every 1–2 min. Synapse software from Tucker Davis Technologies (Alachua, FL, USA) was used to view the neuron firing and the noise levels during the surgery to assure proper placement. An approximate depth of 1900 µm ensured that the length of the electrode containing the 16 contacts spanned throughout the cortex. The craniotomy was then sealed with Kwik-sil (World Precision Instruments, Sarasota, FL, USA ) followed by Teets cold cure dental cement to build a stable headcap on the skull. The skin was sutured together around the electrode using 5-0 monofilament polypropylene suture (Henry Schein, Melville, NY, USA, leaving the head stage open to allow for clipping in for recording. Analgesia and antibiotics (Carprofen, 5 mg/kg and Cefazolin, 25 mg/kg, MWI Veterinary Supple, Boise, ID, USA) were administered for three days post-operatively following surgery.

### 2.2. Electrophysiological Recordings

Electrophysiological recordings from each animal were collected on the day of surgery, the day after surgery, and twice per week thereafter from each animal. All of the animals were tested twice per week, on Tuesdays and Fridays, to maintain consistent spacing between recording days. Animals were exposed to 3% isoflurane to prevent movement during electrode connection. Electrode arrays were connected to a TDT RZ5D BioAmp Processor recording system (Tucker Davis Technologies, Alachua, FL, USA), and the animal was placed in an open box where they were able to move freely while tethered. The experimenter rotated the box on a turntable to force the animal to run and elicit action potentials. All channels were recorded continuously at 24,414 Hz for 10 min for later offline analysis. In the offline analysis, the relative size of the recorded action potentials for each isolatable unit was assessed for each electrode contact based on established methods [42,43]. 

### 2.3. Signal Processing

Signal processing of recorded units was done following previously published methods [12,41,42,43]. In brief, the recorded electrophysiological data was sampled at 24,414 Hz and voltage traces were bandpass filtered at 300–3000 Hz. Using custom MATLAB script, the TDT recorded data were transcribed into MATLAB code so that the signal could then be processed using common average reference. A reviewer blinded to the time point and subject removed artifacts from all recordings to have consistency, using a custom MATLAB script [44]. Spikes were detected when waveforms crossed lower thresholds 3.5 standard deviations of background noise from the mean. Noise was defined as two standard deviations from the mean background noise. The background noise was defined as the median of the absolute deviation of the raw voltage divided by 0.6745 [44]. Signal amplitude was defined as peak-to-peak of that unit’s average waveform. The signal-to-noise (SNR) ratio was calculated using each unit’s signal amplitude divided by the noise calculated for that channel. Single units with a SNR greater than three were included in the analysis. Finally, an unsupervised clustering algorithm, Wave_clus, was used to cluster a minimum of 20 spikes into single units [44]. 

The 10 consecutive channels in layers III and V of the motor cortex were analyzed because the thickness of these cortical layers is approximately 1mm and the electrode contacts on the microelectrode are spaced 100 µm apart. Because the large pyramidal neurons reside in layers III and V of the motor cortex, it was thought that the 10 consecutive channels having the most spiking activity (defined by the highest sum of average units over the 8-week study) were the channels in layers of interest. In cases of recording problems due to water dripping from the animal’s water bottle, bedding and debris in the connector, and/or headstage connector not fitting properly, individual days and/or channels would be removed from analysis. The removed days and channels were randomly distributed across channels, days, and animals’ groups consisting of a total 1.04% of recording days and 1.25% of individual channels. To assess the recording performance over time, the following metrics were calculated from the best 10 consecutive channels: (1) percent of channels detecting single units and (2) number of detectable single units per channel. 

### 2.4. Tissue Processing

Animals were anesthetized with intraperitoneal (IP) injections of ketamine (160 mg/kg) and xylazine (20 mg/kg) (MWI Veterinary Supple, Boise, ID, USA) at the predetermined end point (8 weeks) in preparation for perfusions. Briefly, animals were perfused transcardially with 1X Phosphate Buffer Saline (300 mL or until clear; PBS, Invitrogen, Carlsbad, CA, USA) followed by 30% sucrose (200 mL; Sigma, Burlington, MA, USA) in 1XPBS. The brain was removed from the skull and immediately frozen in optimal cutting temperature compound (OCT, Ted Pella, Redding, CA, USA) on dry ice stored in a −80 °C freezer to be cryosectioned. Brains were sliced transversely in 20 µm sections at −21 °C and mounted on glass slides for downstream histological staining and analysis. The slides were stored at −80 °C until immunohistochemistry.

### 2.5. Immunohistochemistry Histology 

Immunohistochemistry was utilized to quantify the tissue reaction for activated microglia, astrocytes, blood–brain barrier permeability, and total neurons directly around the electrode implant site. Standard immunohistochemistry protocols used in our lab were conducted on the sliced brain tissue [40,41,45]. Briefly, two frozen slides per animal (each containing three tissue slices from layers III and V of the cortex) were equilibrated to room temperature (RT) for one hour. OCT was removed with three 1XPBS washes followed by tissue fixation using 4% formaldehyde for ten minutes at RT and six washes of 1XPBS containing 0.1% Triton-X 100 (Sigma, St. Louis, MO, USA) (1XPBS-T) to rehydrate and permeabilize the tissue. The tissue was blocked for one hour at RT with goat serum blocking buffer (4% *v*/*v* serum (Invitrogen, Carlsbad, CA, USA), 0.3% *v*/*v* Triton-X 100, 0.1% *w*/*v* sodium azide (Sigma, St. Louis, MO, USA). Primary antibodies diluted in goat serum blocking buffer were incubated overnight at 4 °C on the tissue. The primary antibodies used were: mouse antineuronal nuclei (NeuN) (1:250, Millipore, Billerica, MA, USA) for neurons, rabbit antiglial fibrillary acidic protein (GFAP) (1:500, Dako, Santa Clara, CA, USA) for astrocytes, mouse anti-CD68 (ED1) (1:100, Millipore, Billerica, MA, USA) for activated microglia/macrophages, and rabbit anti-immunoglobulin G (IgG) (1:100, AbD Serotec, Hercules, CA, USA) for blood–brain barrier permeability. NeuN and IgG were costained together on the same tissue, while CD68 and GFAP were costained together. NeuN and IgG primary antibodies were incubated separately on different days, with IgG primary antibody on day one and NeuN on primary antibody on day two of staining. The tissue was washed six times with 1XPBT and blocked for 20 min at RT with goat blocking buffer between primary antibody incubations to ensure removal of any unbound primary antibodies. Prior to adding secondary antibodies, the tissue was washed six times with 1XPBS-T for five minutes per wash. Alexa Flour conjugated secondary antibodies (diluted 1:1000 in blocking buffer) were incubated for two hours at RT along with 4’,6-diamidino-2-phenylindole (1:3600, DAPI, Thermo Fisher Scientific, Waltham, MA, USA) to counterstain all cell nuclei. Subsequently, tissue was washed six times with 1XPBS-T and incubated for ten minutes with 0.5 mM copper sulfate buffer (50 mM Ammonium Acetate, pH 5.0) (Sigma, St. Louis, MO, USA) to remove tissue autofluorescence [46]. Slides were washed with multiple distilled water washes prior to mounting with Fluoromount-G (Southern Biotech, Birmingham, AL, USA). An inverted Carl Zeiss AxioObserver Z1 (Carl Zeiss Inc., Thornwood, NY, USA) and AxioCam MRm monochrome camera (Carl Zeiss Inc., Thornwood, NY, USA) was used to image slides using a 10X objective. The MosaiX module in the Zen software was used to take and stitch 16 tile images into one final image, in order to image the entire area around the electrode implant site. All images were imaged using the same optimized exposure times for each of the fluorescent markers. Raw images were exported as 16-bit tagged imaging files (TIFs) for quantitative analysis. 

### 2.6. Quantitative Analysis

Fluorescent intensity profiles around the microelectrode of all cellular markers except for NeuN were analyzed using SECOND, a custom MATLAB program. In brief, tiff images were uploaded into SECOND and the implant site was manually marked. Bins consisting of five µm wide concentric rings, starting from the implant site and spanning out to 1 mm, were defined. The average intensity from 700–750 µm from the implant site was defined as the background and used to normalize the raw fluorescent intensity quantification for each of the markers. The GFAP antibody labels all astrocytes, immature, mature and reactive, thus normalizing the area around the implant site to the background tissue, which allows for the depiction of the reactive astrocytic response around the implanted microelectrode. Following which, the area under the curve in 50 µm increments was obtained using MATLAB and used in statistical analysis [40]. 

Neurons were manually counted using a custom subset of SECOND to quantify neuron populations around the implant site [40]. After the implant site was manually marked, concentric rings up to 600 µm away from the implant site were defined. Neurons were manually counted to obtain the number of neurons per area for each radial distance. The background value was defined as the number of neurons counted in the ring 450–500 µm away from the implant site. This value was used for normalization, thus ensuring that the normalized density was consistent within a cortical layer. Neuron counts were then converted to percentages to the background value. 

### 2.7. Behavior Training and Testing

Behavior practices closely followed previously established protocols [38]. All testing was conducted in a dedicated behavior room with controlled light, sound, and temperature. Animals could become accustomed to the room for 30 min each day prior to behavior testing. Briefly, one week prior to electrode implantation surgery, animals began ladder training and were trained for seven days. Open field grid and grip strength presurgery training was not performed, as these tests do not require learned skill. Animals were tested in the week prior to surgery to establish naïve presurgery performance scores on the open field grid, ladder, and grip strength tests. All postsurgery behavior testing was conducted following the same protocols as presurgery baseline testing, and each animal’s individual postsurgery scores were normalized to their baseline results following Equation (1):

(1) $$\%\;change\;in\;performance=\;\frac{weekly\;test\;score-baseline\;score}{baseline\;score}*\;\left(-100\right)$$

Following electrode implantation, all animals were given a one-week recovery period before beginning postsurgery testing. All of the animals were tested twice per week, on Mondays and Wednesdays, out to eight weeks. Due to the one-week recovery period, the first data point is an average of the testing completed during the second week postimplantation, and data continues out to eight weeks postimplantation.

The open field grid and ladder were manufactured in-house by mechanics at Case Western Reserve University. The open field grid test consisted of a 36 in^2^ acrylic sheet with four opaque walls with a height of 15 in, taped off from the underside into nine equal square sections of 12 in each. Animals could run freely for three minutes, and total distance traveled, number of grid lines crossed, and maximum speed were recorded as metrics of gross motor function. The ladder was 1 m in length and consisted of two clear acrylic walls, 25 cm in height, connected by stainless steel rungs with 3 mm diameter spaced 2 cm apart. The ladder was elevated approximately 20 cm above the ground with a clean cage at the start of the ladder and the animal’s home cage at the finish to encourage completion of the task. Each animal crossed the ladder five times per testing day, and the fastest three completion times and any paw slips were recorded as metrics of fine motor function. The grip strength meter was a standard rodent unit (Harvard Apparatus, Holliston, MA, USA) consisting of a handlebar grip connected to a force meter. Animals could grab the handlebars with both paws and were slowly and consistently pulled away from the meter three times each testing day by the base of the tail until the animal let go. The three scores were averaged as maximum grip strength achieved by each animal. Open field grid and ladder testing was video recorded and analyzed using a custom-developed MATLAB script.

### 2.8. Statistical Analyses

Statistical analyses were performed using statistical software programs Minitab 16 (Minitab Inc., State College, PA, USA) (electrophysiology, histology, and motor behavior group comparisons) and R 3.5.1 with the basic, MASS and “lme4” packages (for regression modeling) as well as the nFCA package built in R interface with Ruby program by Dr. Sun and collaborators (for network analysis) [47]. All sample sizes were an “*n*” of six animals, with analysis being performed as a one per animal basis. 

#### 2.8.1. Statistical Analyses of Electrophysiology, Histology, and Motor Behavior Assessment

Statistical analysis of the two electrophysiological metrics, percent channels recording single units and number of units/channel, were compared within groups by ANOVA with repeated measures to allow for comparisons over time using Minitab 16 (Minitab Inc., State College, PA, USA). Similarly, statistical analysis of the motor behavior metrics, ladder, grid, and grip tests were compared over time within groups by ANOVA with repeated measures. Significance in both of these models was defined as *p* < 0.05. Statistical analysis of the histological metrics, astrocytic scarring, microglia/macrophage activation, and blood–brain barrier permeability utilized the area under the curve for all stains. For group comparisons of neuronal density, the number of neurons per area was used for analysis. Statistical analyses were performed using an ANOVA model in Minitab 16 (Minitab Inc., State College, PA, USA) to allow for comparisons between distances. For significance (defined as *p* < 0.05), Tukey posthoc tests were performed for pairwise comparison. 

#### 2.8.2. Network and Regression Analyses

To understand the relationships the exploratory variables had on the outcome variables, as well as on each other, a network analysis using numerical formal concept analysis (nFCA) was performed [48,49]. nFCA provides an optimal association rule and clustering hierarchy in the logic sense for binary data [48,49]. nFCA developed by the Sun group combined the merit of FCA and statistics to lift out the overall inherent network relations and hierarchical structures from a numerical adjacent/similarity matrix [47,50,51]. It is an unsupervised nonparametric procedure, useful as both an exploratory procedure and a validation procedure for parametric models, such as those regression models in some sense. Because the absolute value of a correlation coefficient (cc) between two variables is between 0 and 1, the smaller a cc is, the weaker the (linear) association between the two variables is. Conversely, a cc close to one would indicate a strong relationship. Thus, we performed nFCA on the matrix of absolute values of correlation coefficients of all variables while keeping the sign of the coefficients in the resulting inherent network structure graphs of all variables from the three metrics. We also performed further statistical analysis to validate the significance of each identified association using an independent bootstrap test to distinguish potentially spurious correlations.

Regression analyses were performed to study and model the association of the behavior and histology metrics with the recording metric. The behavior and histology metrics were considered as surrogates of the functionality of IME. Thus, the two outcome variables in our regression analyses were the number of units per channel (Y1) and the percentage of active channels (Y2). The exploratory variables were the behavioral variables as well as the histology variables measured at one of the two distances, 0–50 µm or 50–100 µm, from the implanted site, with and without being standardized, with an exception for the “grip max force.” “Grip max force” had to be standardized in all cases because its magnitude is vastly different from those of the other variables, leading to fitting errors when not standardized. The candidate models we examined were the generalized linear models (GLM) for Y1 and Y2, negative binomial model as an extension of Poisson model for Y1, and linear mixed effects models (LME) with the transformed outcomes (using the log scale for Y1 and logit scale for Y2), treating the exploratory variables as fixed or mixed effects. The optimal models with the final sets of exploratory variables, for the two outcomes, each with histology measures taken from either of the two distances, were chosen using a combination of Akaike information criterion (AIC), least absolute shrinkage and selection operator (LASSO) and log-likelihood ratio tests. The criterion used in the R software package, “rpql” for LASSO of generalized linear mixed models, is a BIC-type criterion that penalizes a value of log(n) for every nonzero fixed effect coefficient. Similarly, each set of random effects coefficients was penalized a value of log(n) for every nonzero, unique element within the covariance matrix for that set. This combination of penalties was used in the package “lme4”. 

There were a total of four models performed within the regression analyses. Table 1 summarizes the formulas and lists the variables included in each model. There were a total of 480 longitudinal observations included for the “*y*” recording variables in the models, distinguished by the number of measurements taken at each of the 8 time points (weeks 1, 2, 3, 4, 5, 6, 7, and 8) from each of the 10 channels per electrode. There were a total of 54 longitudinal observations included in the “*x*” behavior variables in the models, accounting for the initial baseline measurement as well as the 8 time points measurements taken from each of the 6 animals. Each observation in the longitudinal analysis was not treated as independent. There was one histology measurement taken per animal at the endpoint (8 weeks) at each of the two distance intervals (0–50 µm and 50–100 µm from the implant site) for a total of 6 measurements per model. The histology variables were treated as baseline variables because they were only taken at the endpoint of the study. Treating histology in this manner is similar to how “sex” is included in standard longitudinal analyses. We performed a mixture of longitudinal and cross-sectional data analyses using fixed effects and mixed effects models for each of the four models detailed in Table 1. The intercept refers to the intersection between a linear regression line and the *y*-axis, and the time (*t*) refers to the time variable that is recorded in “weeks”. We chose the best model based on a collective consideration of AIC and conditional R-squared for all of the data. There was only one GLM model utilized due to having a much better AIC value, but very similar conditional R-squared value to the LME model. 

## 3. Results

### 3.1. Electrophysiological Recordings

**Figure 1:**
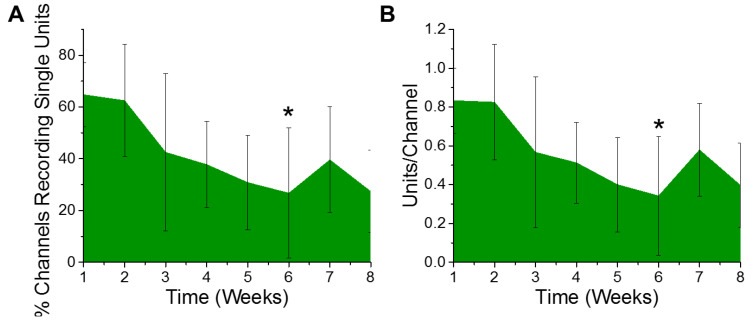
Electrophysiological recordings change over time. There was a significant decrease in recorded (**A**) % channels recording single units (* *p* = 0.05) and (**B**) units per channel between the first- and sixth-week post implantation (* *p* = 0.03). *n* = 6.

Electrophysiological recordings were quantified for the percentage of channels recording, as well as the number of distinct single unit action potentials detected per channel from six animals. Only electrode contacts in layers III through V were included in the analysis, since layer V has the largest neurons and both layers III and V have the majority of the detectible pyramidal neurons. Based on the design of the electrodes used, a total of ten of the sixteen channels were included in the analysis per animal. The ten consecutive channels having the highest average of units per channel over the entire eight week study were chosen as the best ten consecutive channels for that animal. Over the course of the eight week study, we observed a gradual trend of a decreasing percentage of electrode channels detecting single unit activity (Figure 1A). Further, of the electrode contacts that were detecting single unit activity, the number of recordable units per channel demonstrated a similar trend of deceasing over time. However, only the six week time point demonstrated a decrease in the percentage of channels recording single unit action potentials (Figure 1A) or the number of units recorded per channel (Figure 1B), to establish a statistically significant decrease compared to the first week of recording. 

### 3.2. Histology 

**Figure 2:**
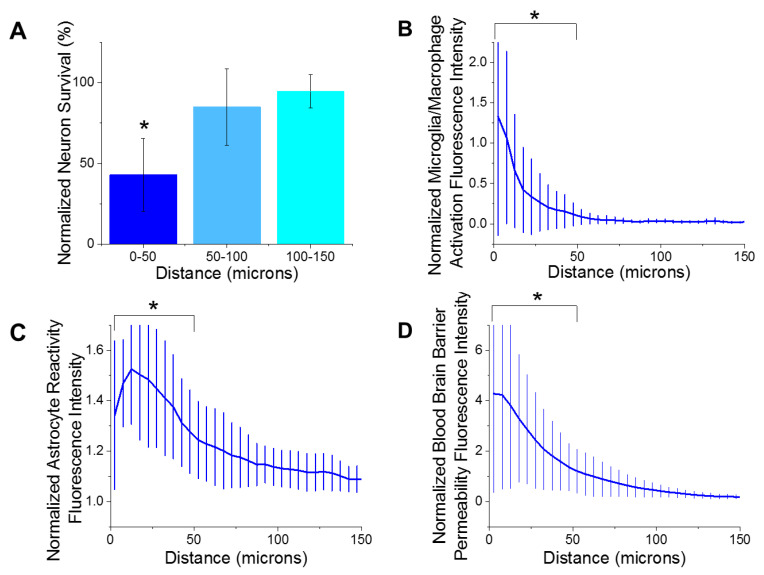
Histological metrics. (**A**) There was a significant decrease (* *p* = 0.000) of neuron density from 0–50 µm distance from the implant site compared to all further distances at 8 weeks post implantation. (**B**) There was significantly more microglia/macrophage activation from 0–50 µm away from the implant site compared to the all further distances (* *p* = 0.004). (**C**) There was significantly more astrocyte reactivity from 0–50 µm away from the implant site compared to the all further distances (* *p* = 0.000). (**D**) There was significantly more blood–brain barrier permeability from 0–50 µm away from the implant site compared to the all further distances (* *p* = 0.003). *n* = 6.

Immunohistological markers for activated microglia/macrophages (CD68), reactive astrocytes (GFAP), blood–brain barrier permeability (IgG), and neuron density (NeuN) were quantified as a function of the distances away from the microelectrode implantation site. The largest emphasis in our analysis was placed on the first 150 µm from the device-tissue interface. It has been widely accepted that neuronal soma must be within 140 µm from the electrode interface in order to distinctly detect single unit action potentials. Further, the first 50 µm is responsible for the highest magnitude action potentials [52]. Our analysis of the histological markers used in the current study demonstrated that there were significantly fewer neurons (Figure 2A) within the first 50 µm of the electrode site, compared to all further distances up to 400 µm. These results are consistent with previous reports from many labs, including our own [29,31,42,53,54,55,56,57]. Additionally, there was significantly more activated microglia/macrophages (Figure 2B), reactive astrocytes (Figure 2C), and blood–brain barrier permeability (Figure 2D) within the first 50 µm of the electrode site compared to further distances. Increased detection of markers for reactive inflammation and breach to the blood–brain barrier are consistent with many previous studies [23,29,37,58].

### 3.3. Motor Function Testing

The three behavior tests utilized in this study each measured a specific aspect of motor function. The open field grid test measures gross motor function [59] and stress behavior [60], while the ladder and grip strength tests measure fine motor function [61,62] and coordinated grasp and muscle function [63], respectively. For all tests, postsurgical performance scores were averaged per week and normalized to animals’ individual presurgery baseline scores, which served as the control levels [38]. In total, six implanted recording animals successfully completed motor function testing and were used in this study. Results are plotted so that a percentage below baseline corresponds to decreased performance, while the closer the percentage gets to baseline indicates an increase in performance. All errors are reported as standard error of the mean (SEM).

**Figure 3:**
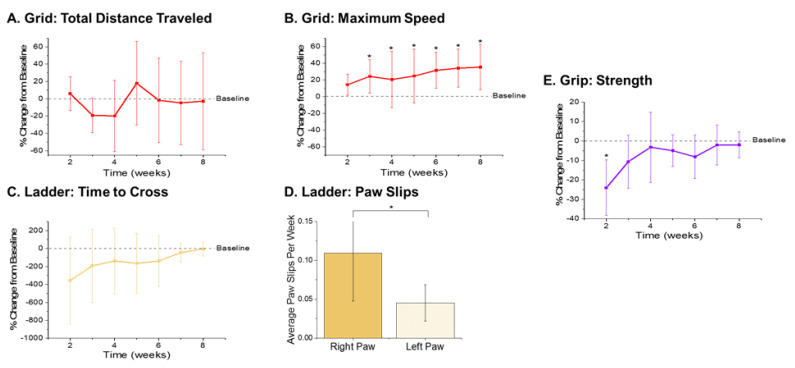
Motor Behavior Metrics. Gross motor function metrics from the open field grid test revealed (**A**) no significant differences in the distance traveled and (**B**) significantly higher maximum velocity achieved in postsurgical weeks 3–8 (*p* = 0.035, 0.007, 0.009, 0.007, 0.004, and 0.003, respectively) compared to baseline performance. Fine motor function and grasp were assessed via ladder and showed an (**C**) initial decrease in time to cross the ladder followed by gradual increase back to baseline by 8 weeks postimplantation, and (**D**) a significant increase in the average number of right paw slips compared to left paw slips (*p* = 0.008). Secondary assessment of fine motor function and grip was measured using a grip strength meter and showed (**E**) a significant decrease in grip strength in the first postsurgical testing week (*p* = 0.003). *n* = 6.

The metrics recorded from the open field grid test were the total distance traveled and maximum speed achieved (Figure 3A,B). There were no significant differences observed with the total distance traveled during any of the times points compared to baseline performance (Figure 3A). However, there were significantly higher velocities observed during weeks 3–8 postsurgery compared to baseline performance (Figure 3B). The increased velocities observed herein may be indicative of a practice effect, given that our previously published findings observed increased performance on the grid test with the control nonimplanted group as well as the implanted group [38].

The metrics quantified from the ladder test were the time it took each animal to cross the ladder and the number of slips from each of their front paws. As compared to baseline performance, there were some statistically nonsignificant changes observed that are worth noting. There was an initial decrease in the performance of the animals, indicated by a large increase in the time it took to cross the ladder (Figure 3C). Interestingly, a gradual increase in performance times was observed, with animals recording times close to baseline by eight weeks postimplantation (Figure 3C). This increase back to baseline is unlike our previously published findings, which presented a steady decrease in ladder crossing performance up to 16 weeks. The dissimilarities between the two study findings are attributed to the distinctions between the experimental set-ups, including the type of electrode implanted, head cap size, and frequency of animal handling. For example, in this correlation study, animals were handled an extra two days per week for the awake, freely moving electrophysiological recordings. The increased handling, social interaction, and opportunity to run around during electrophysiological recordings may have facilitated the development of improved motor skills, thus resulting in faster ladder crossing times during this study [64]. Because the electrodes were always implanted into the left hemisphere, the number of paw slips from the left and right side were also compared to assess fine motor skills. Notably, there were close to zero right or left paw slips from any of the animals prior to surgery (Supplementary Figure S3). There was a significant increase in the average number of paw slips per week observed from the right paw compared to left paw (Figure 3D), which coincides with our previously published findings [38]. 

As a secondary measure of fine motor function and coordinated grasp, maximum grip strength was recorded for each animal. There was a significant reduction of grip strength at the first postsurgical time point compared to baseline observed in animals implanted with electrodes (Figure 3E). However, grip strength recovered considerably after the second week postimplantation and all other postsurgical time points did not exhibit any significant differences between the baseline and postsurgical performance.

### 3.4. nFCA Network Relationships between Recording, Motor Behavior and Histology

**Figure 4:**
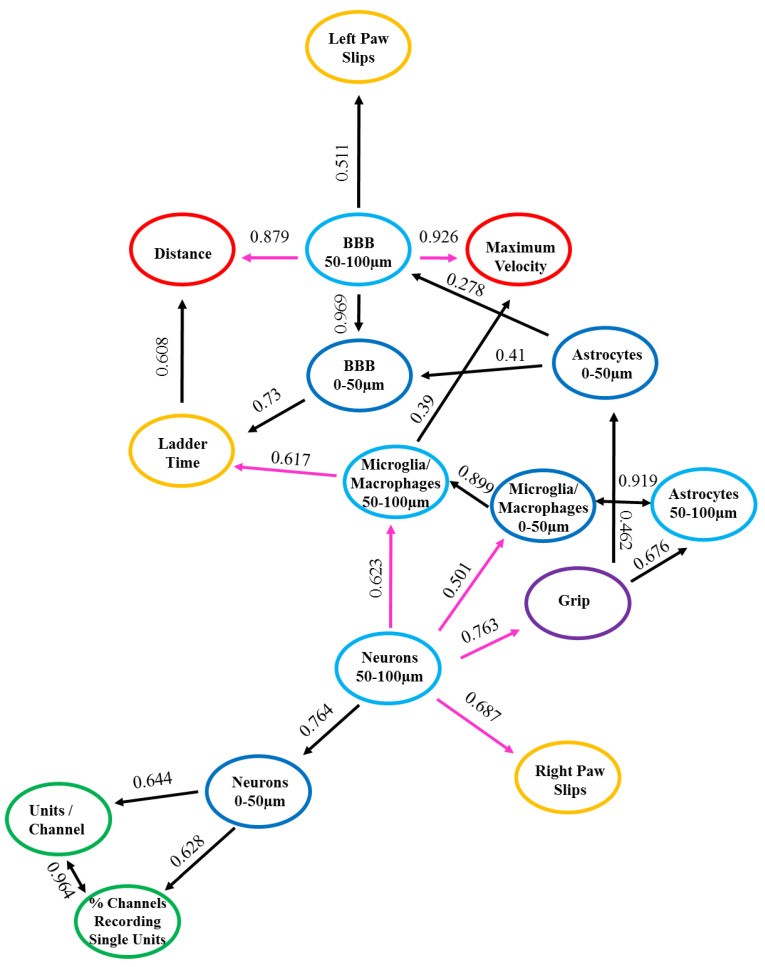
Associations with Positive and Negative Relationships. Graphical representation depicting the positive (in black color) and negative (in magenta color) relationships between the measured variables. *n* = 6.

In order to assess potential correlative relationships between the recording, behavior, and histological data, a type of cause mediation network analysis, nFCA, was performed. The nFCA analysis produces a numerical concept analysis, which shows the overall associations of the corresponding variables (“*x*” and “*y*”) in the population we studied, with lines and strengths lifted out from all pairwise relationships, using an optimization algorithm. The nFCA analysis considers the associations of all variables (including the associations between each “*x*” and “*y*”, and between all “*x*” variables) and builds an ‘optimal’ network structure of these associations by lifting out the strong associations between all “*x*” variables, and between each of “*x*” with “*y*”, using lines and strength indicators shown in Figure 4. nFCA is analogous to providing an aerial view of an “entire forest” with zoomed-in details of important relations, based on the simple neighboring relationships that one can only see when restricted locally. Hence, the variable with more connected lines and larger association values on these lines has stronger and more significant relationships than its neighboring variables are with any other variables, and is considered a potential ‘dominant’ variable that can provide new insights. The arrow in our network analysis indicates a stronger relationship of the variable at the tail end with all other variables (other “*x*” or “*y*”), than the variable at the arrow tip with all other variables. The direction of the arrow projection acknowledges which variable is the dominating variable. The ‘dominator’ or a ‘dominance’ relationship is defined as having a more and/or stronger significant association than any other variables are with their neighboring variables, including the outcome variable. The lines with two arrowheads depict bidirectional relationships, indicating that the variables are associated to one another.

The numbers along the lines signify the strength of the relationship between the two variables. The closer the number is to one, indicates that relationship is stronger compared to those closer to zero. The length of the lines depicts the proximity of the relationship, denoting that the shorter the line is, the closer the relationship is between the two variables. The strengths of these lines are the values represented in Table 2 and Figure 4. It is important to note that although the dominant and strong relationships may be numerically significant, they may not all be biologically relevant. Therefore, the biological relationships are the biologically meaningful relationships arising from the data. We compared the outcomes from the network analysis to the regression analysis to clarify their complementary implications and perspectives. These perspectives, combined with meaningful biological interpretations, provide new insights and set a basis for future biologically-relevant studies. The biological relationships will be the data presented with an interpretation and discussed below. 

The nFCA shows a network of connections between the recording, behavior, and histology variables with positive and negative associations, and sometimes no associations at all between the variables studied. A positive association indicates that the variables have a direct relationship, meaning that when the one variable increases, the other variable(s) will also increase. Whereas, a negative relationship indicates that variables have an inverse relationship, signifying that when one variable decreases, the other variable(s) will then increase. Table 2 and Figure 4 list and illustrate the results from the nFCA, respectively, both of which display the same data but in different formats for readability. The colors from Figure 4 correspond to those in Table 2, with black lines depicting positive associations, pink lines as the negative associations, and the colored circles representing the variables utilized in the nFCA model: recording in green, ladder test in orange, grip in purple, grid in red, histological outcomes from 0–50 µm from the electrode interface in dark blue, and the 50–100 µm distance intervals in light blue. The positive and negative relationships between the measured variables are summarized in Table 2 and illustrated in Figure 4. The variables depicting direct and inverse associations, respectively, as well as a biological relationship between one another are detailed and discussed below.

#### 3.4.1. Positive Relationships

The electrophysiology metrics, recordable units per channel and percentage of channels recording single units, had bidirectional positive relationships (0.964) with each other (Table 2 and Figure 4). This bidirectional positive relationship indicates that when there is an increase in percentage of channels recording single units there may also be an increase in the number of units recorded per channel. The neuronal density at the 0–50 µm distances from the implant site had a positive relationship, which was directly associated to the electrophysiology metrics, recordable units per channel (0.644) and percentage of channels recording single units (0.628) (Table 2 and Figure 4). 

The only behavior metrics displaying positive relationships between each other were the time it took to cross the ladder test and the distance travelled in the grid test (0.608) (Table 2 and Figure 4). Interestingly, there was a direct association between the histological measurement for blood–brain barrier (BBB) permeability at 0–50 µm from the implant site and the time it took to cross the ladder test (0.73) (Table 2, Figure 4). The BBB permeability at 50–100 µm from the implant site displayed a positive relationship to the number of left paw slips from the ladder test (0.511) (Table 2 and Figure 4). The latter two observations indicate that the increase of BBB permeability may be associated with the decrease in performance on the ladder test. 

There were positive relationships between the histological measurements for activated microglia/macrophages at 0–50 µm distance to the microglia/macrophages 50–100 µm distance (0.899), astrocyte reactivity at 0–50 µm (0.873), and 50–100 µm (0.919) distances, demonstrating the direct associations these glial cells have on one another closest to the electrode tissue interface (Table 2 and Figure 4). 

#### 3.4.2. Negative Relationships

The neuronal density at 50–100 µm was inversely associated with the outcome of motor behavior tests, including the grip test (0.763) and the right paw slips from the ladder test (0.683) (Table 2 and Figure 4). Moreover, there were negative relationships between the neuronal density at 50–100µm and the microglia/macrophage activation at 0–50 µm (0.501) and 50–100 µm (0.623) distances from the implant site (Table 2 and Figure 4). The BBB permeability at the 50-100 µm distance interval had an inverse association with the distance traveled (0.879) and the maximum velocity (0.926) during the grid test (Table 2 and Figure 4). Finally, the microglia/macrophage activation at 50–100 µm distance interval had an inverse relationship with the time it took to cross the ladder test (0.617) (Table 2 and Figure 4). 

### 3.5. Correlations between Recording, Motor Behavior, and Histology

The above nFCA analysis describes the overall relationships between the variables when they are all interacting together. To better understand the relationships between the behavior and histology variables with the electrophysiology recording data, pairwise correlations using the linear mixed effects (LME) model and the generalized linear model (GLM) were run. The estimated correlation coefficient of a given variable within the regression models accounts for the additive effects of other variables that are included in the model, whereas the nFCA models do not. The significance of a variable’s association implied by a regression analysis relies on if the exploratory variable “*x*” is selected into a subset of all possible predictive factors. It is extremely important to consider multiplicity and spurious events in a regression analysis. When considering all predictor exploratory variables “*x*” together in a multiple regression for building a predictive model for the outcome variable “*y*”, sometimes including all of the exploratory predictor variables did not improve our knowledge about the outcome variable “*y*”. Therefore, the LME and GLM models only applied the exploratory variables that passed the AIC, LASSO, and log-likelihood ratio tests. Thus, the subset chosen for our analysis provided the best predictive set of variables for modeling the outcome variable “*y*” regardless of the association of the exploratory variable “*x*” with other possible predictive variables. Correlating the outcome variables to the percentage of channels recording single units demonstrated significant correlations between the variables. The findings from assessing the correlations using the LME model and the GLM were comparable, thus only the LME model will be discussed here (Table 3).

When considering only the histology outcome measurements at 0–50 µm distance from the implant site, together with the longitudinal variables, it was found that the maximum velocity from the grid test (*p* = 0.003), the blood–brain barrier (BBB) permeability (*p* = 3.91 × 10^−8^), microglia/macrophage activation (*p* = 6.31 × 10^−6^), and astrocyte reactivity (*p* = 9.48 × 10^−6^) were significantly correlated to the percentage of channels recording single units. A similar model using the histology outcome measurements at 50–100 µm distance found that the maximum velocity from the grid test (*p* = 0.001), the time during the ladder test (*p* = 0.02), and the blood–brain barrier (BBB) permeability (*p* = 0.03) were significantly correlated to the percentage of channels recording single unit action potentials. 

When examining the histology outcome measurements at 0–50 µm distance from the implant site, together with the longitudinal variables, it was found that there were significant correlations between the number of units recorded per channels to the right paw slips from the ladder test (*p* = 0.015), the maximum velocity from the grid test (*p* = 0.006), the BBB permeability (*p* = 2.51 × 10^−6^), microglia/macrophage activation (*p* = 4.58 × 10^−6^), and astrocyte reactivity (*p* = 9.57 × 10^−7^). Similarly, when evaluating the same model using the histology at 50–100 µm distance from the implant, it was found that there were significant correlations between the number of units recorded per channels to the time during the ladder test (*p* = 0.05) and the maximum velocity during the grid test (*p* = 0.01). 

Collectively, these results indicate that the outcome variables with significant correlations have an association of contribution explaining the response variable, which may have a positive or negative influence on the outcome of the units recorded per channel. 

## 4. Discussion

The overarching goal of this study was to utilize novel nFCA analysis as well as traditional regressions analyses to evaluate the relationships between electrophysiological recordings from intracortical microelectrodes and the histological outcomes, as well as observed functional deficits from the associated cortical region. Because the electrodes were implanted in the motor cortex in this study, behavioral tests evaluating the motor function performance were utilized as a metric to further assess electrode performance and biological response. Studies implanting electrodes in other regions of the brain can utilize numerous other behavioral analyses to extend evaluation of electrode implementation. Here, the same group of animals were evaluated over eight weeks for quality and stability of electrophysiological recordings, by quantifying the percentage of channels able to record units and the number of units recorded per channel. Although, measurements of signal-to-noise ratio (SNR), amplitude, and noise have been commonly used metrics in assessment of recording quality, it has been recently demonstrated that perhaps SNR may be less informative, due to the covariance of amplitude and noise [57]. The motor behavior was also assessed during the eight weeks, by repeated tests measuring the associated fine and gross motor function through performance during an open field grid test, ladder test, and grip strength tests, compared to baseline performances recorded prior to IME implantation. Postmortem histological findings for neuronal density, blood–brain barrier permeability, activation of microglia/macrophages, and reactive astrocytes were evaluated. The relationships between these variables were dissected utilizing advanced nFCA network analysis and indepth correlative analysis. This initial nFCA network analysis revealed complimentary relationships between the variables. 

While there have been some studies performed to relate histological data to recording performance [10,24,25,59,65,66], these studies do not take account of the relationships between these variables to the functional performance of the corresponding region of the brain, nor do they evaluate how all of these variables are associated to one another when they are acting together. Moreover, correlative studies assessing histological outcomes and recording performance have observed the relationships between only a subset of variables, one at a time [57,66]. Our more complex statistical analysis found that BBB disruption has a direct association with recording quality and stability, as well as motor function. BBB disruption has been implicated with affecting the electrode–tissue interface and recording stability [37,59]. Recent studies evaluating the molecular pathways following electrode implantation have further implicated the role BBB disruption has on exposing the brain parenchyma to pro-oxidants, pro-inflammatory factors, and blood-borne molecules [21,67,68]. The entrance of such toxins to the brain parenchyma, through BBB disruption, can be linked to neurological damage, as BBB disruption underlies many neurodegenerative neuropathologies [69]. Our group has previously shown a correlation with BBB permeability and decrease in motor behavior performance following electrode implantation, specifically the time it took to complete the ladder test [38]. Similarly, the current study found an association between BBB permeability and the time it took to complete the ladder test, as well as the maximum velocity and the distance traveled during the grid test. 

It has been documented that single unit recording occurs from the neurons closest to the electrode site, spanning up to 140 µm away from the recording sites, with the signal amplitude depreciating with increasing distance [53]. Notably, neuronal density showed a direct relationship to recording quality, depicted by the positive associations with both of the electrophysiological metrics evaluated. Moreover, neuronal density had an inverse relationship with the strength of the animals during the grip test and the number of times their paw slipped during the ladder test. These findings indicate that there is a relationship between the loss of neurons around the electrode and the observed decrease in motor behavior assessments, such as fine motor deficits and reduction of animal’s grip strength. Further experimental analysis must be conducted in order to accurately decipher and understand this relationship. Previous work from our lab has shown motor deficit post electrode implantation, but attributed this deficit to only the BBB disruption. The type of correlative analysis performed in the study herein took into account additional variables utilized to investigate the biological response and electrode functionality, which were not included in our aforementioned study. The inclusion of these variables allowed for the advanced correlation assessments presented in this study, which revealed additional information regarding the relationships between the variables investigated. It must be emphasized that the relationships implicated in the current report are only generalizable to the situations that are similar to ours. Its generalization to a broad setting must consider experimental variability across studies, experimental preparations over time, and between research laboratories. The novelty is in our bidirectional approach, using both regression modeling and network analysis. Examining complex associations from multiple angles is a paradigm change to a stand-alone simple regression analysis; showing new insights from multiple angles and measures are particularly valued in this emerging field of neural engineering [70]. Specifically, the benefits and advantages of nFCA analysis lies within the ability to study the relationships between the numerous diverse variables utilized within one study at once. nFCA shows the overall relationships between the corresponding variables while they are all interacting together. Furthermore, the regression analyses in our study were also more complex than simpler regression analysis due to the use of AIC, LASSO, and log-likelihood analyses. These analyses were used to inform the appropriate variables to include in the correlation models to achieve the best predictive set of variables for modeling the outcome variables.

The implantation of IME has been described to create a “dead zone” around the implant, due to the inflammatory molecules released from activated glial cells, thus causing the death and degeneration of neurons nearby [18,20,23,29,30,55]. Correspondingly, the glial cell response had associations with one another, distinguished by activated microglia/macrophages and reactive astrocytes connections and formed correlative relationships. Glial cells can perpetuate the inflammatory response to implanted electrodes by signaling the presence of damage through the release of pro-inflammatory factors, barricading the electrode by building a glial scar, and damaging the neurons by releasing neurotoxic factors such as reactive oxygen species [13,15,18,19,20,21,29,43]. Expectedly, there is an inverse relationship between the glial cells response and the neuronal density, implying, as there are more glial cells’ activation, there is a decrease in neuronal populations and vice versa. Glial cell reactivity has been reported to be correlated with signal amplitude recorded immediately after implantation (three days post implant) [66]. Given that the cells in the brain communicate with one another and work together to function, it is no surprise that the collective microglia/macrophage, astrocyte, and neuron response influences the recording performance evaluated from the implanted intracortical microelectrodes. 

It is critical to include all of the evaluated variables in an nFCA network assessment in order to understand the association of all of these variables collectively with one another. On the other hand, when investigating the effects certain variables have on one specific outcome measurement, such as the number of units recorded per channel, it is valuable to understand how to choose the right variables to incorporate into the correlation model to measure the appropriate correlations. Here we utilized AIC, LASSO, and log-likelihood analyses to inform the appropriate variables to include in the correlation models. In contrast to recent findings of no histological correlations with electrophysiological recordings, this study displayed some relationships between the variables investigated [57]. When observing only the distance interval 0–50 µm away from the implant site, it was found that microglia/macrophage and astrocyte reactivity, BBB disruption, and maximum velocity during the grid test were all significantly correlated to the electrophysiological outcomes measured (units/channel and percentage of channels recording single units). These results coincide with the nFCA analysis, which incorporated all of the measured variables at once, emphasizing the role glial cell activation, BBB disruption, and motor performance have on recording quality. 

## 5. Conclusions

The objective of this pilot study was to utilize novel numerical FCA network analysis and traditional correlation regression analyses to evaluate the relationships between electrophysiology, histology, and motor performance metrics. The analyses discovered complimentary relationships between the variables. The histological variables for glial cell activation had associations between each other, as well as the neuronal density around the electrode interface. The neuronal density had associations to the electrophysiological recordings and some of the motor behavior metrics analyzed. Although there are some limitations to correlative analysis, it is a valuable tool to assess and understand the relationships between the diverse variables being investigated. These approaches can be further utilized to improve the understanding of the relationships between various other metrics to enhance the recognition of the factors associated to the chronic decrease in IME performance. Further investigation utilizing histological and gene expression metrics for oxidative stress, inflammation, and neuronal health can help decipher the relationship between neural cells and device functionality. The models presented here can also be applied to ongoing investigations, utilizing various configurations of devices and therapeutics, mechanical stability, and material failure, to evaluate the relationships between those confounding variables to the inflammatory response, electrophysiology recordings quality, and motor behavior function. 

## Figures and Tables

**Table 1:** Details of the regression analyses models. The formulas depict the relationships between the longitudinal versus single time point variables. Although there were only *n* = 6 animals, there was a total 480 observations for the “*y*” recording outcome variables, 54 observations for the “*x*” behavior variables, and 6 observations for each of the “*x*” histology variables per distance interval.

	Outcome Variable: Percent Channels Recording Single Units		Outcome Variable: Units/Channel
	AIC Value: 1756.784 LME model with random intercept and slope		AIC Value: 619.05 GLM model
**0–50 µm Histology Distance**	logit(***y*** (*t*)) ~ ***b*** × ***x*** + *a*1_i_ + *a*2_i_ × time, where “i” is the ith subject, ***b*** = (*b*_0_, *b*_1_, …, *b*_q_) and ***a*_i_** is the random effects ***y*** = % channels recording single units ***x*** = (1, time, Right Paw Slip(*t*), Left Paw Slip(*t*), Maximum Velocity(*t*), BBB Permeability, Microglia/Macrophage Activation, Astrocyte Reactivity)		log(***y*** (*t*)) ~ ***b*** × ***x***, where “i” is the ith subject, ***b*** = (*b*_0_, *b*_1_, …, *b*_q_) ***y*** = units/channel ***x*** = (1, Right Paw Slip(*t*), Maximum Velocity(*t*), BBB Permeability, Microglia/Macrophage Activation, Astrocyte Reactivity)
**50–100 µm Histology Distance**	AIC Value: 1768.454 LME model with random intercept		AIC Value: 1136.184 LME model with random intercept
	logit(***y*** (*t*)) ~ ***b*** × ***x*** + *a*_i_, where “i” is the ith subject, ***b*** = (*b*_0_, *b*_1_, …, *b*_q_) and *a*_i_ is the random effect ***y*** = % channels recording single units ***x*** = (1, Ladder Time(*t*), Left Paw Slip(*t*), Maximum Velocity(*t*), BBB Permeability, Neuron Density)		log(***y*** (*t*)) ~ ***b*** × ***x*** + *a*_i_, where “i” is the ith subject, ***b*** = (*b*_0_, *b*_1_, …, *b*_q_) and *a*_i_ is the random effect ***y*** = units/channel ***x*** = (1, Ladder Time(*t*), Right Paw Slip(*t*), Left Paw Slip(*t*), Maximum Velocity(*t*), BBB Permeability, Neuron Density)

**Table 2:** nFCA Relationships between all Measured Variables. The table below depicts the 16 variables investigated in this study within the three metrics of interest: recording, behavior, and histology. The type of relationship (i.e., positive or negative), number of networks and specific network variables associated, and the strength and direction of that relationship are all summarized below. The color scheme in the table is uniform to that of Figure 4. *n* = 6.

	nFCA Relationships					
	Variable	Relationship	# Networks	Network Variable	Strength	Direction
**Recording Metrics**	**% Channels Recording Single Units**	Positive	1	**Units/Channel**	0.964	BI
	**Units/Channel**	Positive	1	**% Channels Recording Single Units**	0.964	BI
**Behavior Metrics**	**Maximum Velocity (Grid)**	N/A	0	**N/A**	N/A	N/A
	**Distance (Grid)**	N/A	0	**N/A**	N/A	N/A
	**Ladder Time**	Positive	1	**Distance (Grid)**	0.608	UNI
	**Left Paw Slip (Ladder)**	N/A	0	**N/A**	N/A	N/A
	**Right Paw Slip (Ladder)**	N/A	0	**N/A**	N/A	N/A
	**Grip**	Positive	1	**Astrocyte Reactivity (0-50 µm)**	0.462	UNI
		Positive		**Astrocyte Reactivity (50-100 µm)**	0.676	UNI
**Histology Metrics**	**Neurons (0–50 µm)**	Positive	2	**% Channels Recording Single Units**	0.628	UNI
		Positive		**Units/Channel**	0.644	UNI
	**Neurons (50–100 µm)**	Positive	5	**Neurons (0–50 µm)**	0.764	UNI
		Negative		**Right Paw Slip (Ladder)**	0.683	UNI
		Negative		**Grip**	0.763	UNI
		Negative		**Microglia/Macrophages Activation (0–50 µm)**	0.501	UNI
		Negative		**Microglia/Macrophages Activation (50–100 µm)**	0.623	UNI
	**Blood–Brain Barrier (BBB) Permeability (0–50 µm)**	Positive	1	**Ladder Time**	0.73	UNI
	**Blood–Brain Barrier (BBB) Permeability (50–100 µm)**	Positive	4	**Blood–Brain Barrier (BBB) Permeability (0–50 µm)**	0.969	UNI
		Positive		**Left Paw Slip (Ladder)**	0.511	UNI
		Negative		**Maximum Velocity (Grid)**	0.926	UNI
		Negative		**Distance (Grid)**	0.879	UNI
	**Microglia/Macrophages Activation (0–50 µm)**	Positive	3	**Microglia/Macrophages Activation (50–100 µm)**	0.899	UNI
		Positive		**Astrocyte Reactivity (0–50 µm)**	0.873	UNI
		Positive		**Astrocyte Reactivity (50–100 µm)**	0.919	BI
	**Microglia/Macrophages Activation (50–100 µm)**	Positive	2	**Maximum Velocity (Grid)**	0.39	UNI
		Negative		**Ladder Time**	0.617	UNI
	**Astrocyte Reactivity (0–50 µm)**	Positive	2	**Blood–Brain Barrier (BBB) Permeability (0–50 µm)**	0.41	UNI
		Positive		**Blood–Brain Barrier (BBB) Permeability (50–100 µm)**	0.278	UNI
	**Astrocyte Reactivity (50–100 µm)**	Positive	1	**Microglia/Macrophages Activation (0–50 µm)**	0.919	BI

**Table 3:** Estimated Coefficients and Significance of Exploratory Variables. The table below portrays the variables included in the pairwise correlations using the linear mixed effects models and their resulting estimates and p-values in relation to either the percentage of channels recording single units or the units per channel outcome variables. *n* = 6.

	Outcome Variable: Percent Channels Recording Single Units				Outcome Variable: Units/Channel		
	Exploratory Variable	Estimate	*p*-value		Exploratory Variable	Estimate	*p*-value
**0–50 µm Histology**	Right Paw Slip (Ladder)	−1.955	0.146		Right Paw Slip (Ladder)	−1.390	0.015
	Left Paw Slip (Ladder)	−4.348	0.124		Maximum Velocity (Grid)	−3.580	0.006
	Maximum Velocity (Grid)	−11.760	0.003		BBB Permeability	−0.029	2.51 × 10^−6^
	BBB Permeability	−0.082	3.910 × 10^−8^		Microglia/Macrophage Activation	−0.205	4.58 × 10^−6^
	Microglia/Macrophage Activation	−0.550	6.310 × 10^−6^		Astrocyte Reactivity	0.520	9.57 × 10^−7^
	Astrocyte Reactivity	1.260	9.480 × 10^−6^		Intercept	0.413	0.510
	Intercept	4.724	0.008				
	Time	−0.215	0.240				
**50–100 µm Histology**	Ladder Time	0.019	0.020		Ladder Time	0.007	0.054
	Ladder_Left_Front_Slips	−0.127	0.967		Right Paw Slip (Ladder)	−0.960	0.100
	Maximum Velocity (Grid)	−12.072	0.001		Left Paw Slip (Ladder)	−0.833	0.500
	BBB Permeability	−0.303	0.027		Maximum Velocity (Grid)	−3.525	0.013
	Neuron Density	0.026	0.245		BBB Permeability	−0.091	0.088
	Intercept	3.448	0.149		Neuron Density	0.006	0.528
					Intercept	0.446	0.661

## Supplementary Materials

The following are available online at https://www.mdpi.com/2072-666X/11/9/838/s1, Tables detailing each animal’s electrophysiological, motor behavior, and histological results, including the mean and standard deviation for each time point. Figure S1: Depiction of each animals’ electrophysiological performance metrics individually plotted. Figure S2: Graphical representation of each animal’s performance on the Ladder test. The graph on the left represents each animal’s time to cross the ladder, while the graph on the right illustrates the normalized percent change in time to cross the ladder as compared to baseline. The graph on the left demonstrates the percentage of working channels recording single unit activity and the graph on the right illustrates the average single units recorded per channel. Figure S3: Number of paw slip from each animal. The left graph represents the right paw slips and the right graph represents the left paw slips.
