# Supplementary material for: Investigating the Association between Motor Function, Neuroinflammation, and Recording Metrics in the Performance of Intracortical Microelectrode Implanted in Motor Cortex
Source: Micromachines (Basel). 2020 Sep 3;11(9):838. doi: 10.3390/mi11090838 (PMC7570280; doi:10.3390/mi11090838)
Supplement: Supplementary file 1 [file micromachines-11-00838-s001.pdf]

micromachines

MDPI

Investigating the Association between Motor Function, Neuroinflammation, and Recording Metrics in the Performance of Intracortical Microelectrode Implanted in Motor Cortex

Evon S. Ereifej, Youjun Li, Monika Goss-Varley, Youjoung Kim, Seth M. Meade, Keying Chen, Jacob Rayyan, He Feng, Keith Dona, Justin McMahon, Dawn Taylor, Jeffrey R. Capadona and Jiayang Sun

Tables detailing each animal's electrophysiological, motor behavior, and histological results, including the mean and standard deviation for each time point.

Electrophysiology: Percentage of Channels Recording Single Units								
Time (weeks)	Animal Name						Average	SD
	CR1	CR2	CR3	CR4	CR5	CR6		
1	66.67	56.67	66.67	45.00	78.52	75.00	64.75	12.31
2	40.00	35.00	90.00	75.00	75.00	60.00	62.50	21.62
3	15.00	5.00	75.00	40.00	80.00	40.00	42.50	30.45
4	35.00	35.00	25.00	45.00	66.67	20.00	37.78	16.62
5	45.00	15.00	20.00	15.00	60.00	30.00	30.83	18.28
6	35.00	0.00	10.00	50.00	60.00	5.56	26.76	25.14
7	25.00	10.00	45.00	65.00	57.22	35.56	39.63	20.45
8	40.83	5.00	23.33	44.44	36.94	13.70	27.38	15.92

Electrophysiology: Units/Channel								
Time (weeks)	Animal Name						Average	SD
	CR1	CR2	CR3	CR4	CR5	CR6		
1	1.17	0.80	0.93	0.70	0.89	0.75	0.87	0.17
2	0.50	0.45	1.20	0.90	1.05	0.85	0.83	0.30
3	0.20	0.05	1.00	0.70	0.95	0.50	0.57	0.39
4	0.40	0.65	0.35	0.65	0.78	0.25	0.51	0.21
5	0.55	0.15	0.20	0.30	0.80	0.40	0.40	0.24
6	0.55	0.00	0.15	0.65	0.65	0.06	0.34	0.31
7	0.45	0.30	0.70	0.95	0.67	0.41	0.58	0.24
8	0.56	0.08	0.53	0.47	0.57	0.17	0.40	0.22

Motor Behavior: Ladder Time								
Time (weeks)	Animal Name						Average	SD
	CR1	CR2	CR3	CR4	CR5	CR6		
**Baseline**	8.98	3.80	12.63	5.16	24.83	3.49	9.82	8.15
**2**	108.54	3.67	47.34	9.29	95.77	4.14	44.79	47.48
**3**	108.43	4.79	27.31	9.23	18.97	3.76	28.75	40.07
**4**	96.77	5.24	10.92	9.86	14.34	3.80	23.49	36.10
**5**	85.28	4.91	6.96	12.03	43.07	4.13	26.06	32.51
**6**	77.11	4.94	10.56	14.03	31.34	3.19	23.53	28.10
**7**	31.01	3.85	18.67	11.46	16.27	3.93	14.20	10.26
**8**	20.20	2.62	19.32	6.60	9.26	3.88	10.31	7.67

Motor Behavior: Ladder Right Paw Slips								
Time (weeks)	Animal Name						Average	SD
	CR1	CR2	CR3	CR4	CR5	CR6		
**Baseline**	0.00	0.00	0.00	0.00	0.00	0.20	0.03	0.08
**2**	0.20	0.10	0.20	0.11	0.00	0.33	0.16	0.11
**3**	0.00	0.40	0.10	0.20	0.10	0.20	0.17	0.14
**4**	0.00	0.10	0.20	0.20	0.00	0.00	0.08	0.10
**5**	0.20	0.40	1.00	0.00	0.00	0.00	0.27	0.39
**6**	0.10	0.10	0.20	0.00	0.00	0.00	0.07	0.08
**7**	0.10	0.00	0.30	0.00	0.00	0.00	0.07	0.12

Motor Behavior: Ladder Left Paw Slips								
Time (weeks)	Animal Name						Average	SD
	CR1	CR2	CR3	CR4	CR5	CR6		
**Baseline**	0.00	0.00	0.00	0.00	0.00	0.00	0.00	0.00
**2**	0.20	0.00	0.00	0.00	0.05	0.05	0.05	0.08
**3**	0.10	0.10	0.20	0.00	0.00	0.00	0.07	0.08
**4**	0.10	0.00	0.10	0.00	0.00	0.00	0.03	0.05
**5**	0.10	0.00	0.10	0.00	0.10	0.10	0.07	0.05
**6**	0.20	0.10	0.10	0.00	0.00	0.00	0.07	0.08
**7**	0.00	0.10	0.00	0.00	0.10	0.00	0.03	0.05

Motor Behavior: Grid Total Distance Traveled								
Time (weeks)	Animal Name						Average	SD
	CR1	CR2	CR3	CR4	CR5	CR6		
**Baseline**	9.63	11.54	13.65	11.78	13.61	15.13	12.56	1.96
**2**	7.20	13.90	12.26	14.90	15.17	17.07	13.42	3.43
**3**	5.69	12.57	12.56	10.06	7.75	12.41	10.17	2.91
**4**	0.93	15.03	13.24	11.45	11.70	9.19	10.26	4.97
**5**	3.79	17.76	15.33	21.32	15.50	16.17	14.98	5.91
**6**	1.79	14.40	13.73	19.49	13.06	12.69	12.53	5.81
**7**	0.82	16.34	10.93	15.53	12.89	17.33	12.31	6.10
**8**	0.87	17.75	11.77	17.07	17.52	9.17	12.36	6.64

Motor Behavior: Grid Maximum Velocity								
Time (weeks)	Animal Name						Average	SD
	CR1	CR2	CR3	CR4	CR5	CR6		
**Baseline**	0.32	0.29	0.34	0.36	0.30	0.38	0.33	0.04
**2**	0.36	0.36	0.35	0.38	0.40	0.41	0.38	0.03
**3**	0.35	0.39	0.39	0.43	0.48	0.40	0.41	0.04
**4**	0.19	0.45	0.44	0.48	0.42	0.41	0.40	0.10
**5**	0.22	0.45	0.45	0.46	0.46	0.43	0.41	0.10
**6**	0.32	0.45	0.40	0.49	0.45	0.50	0.43	0.07
**7**	0.34	0.44	0.40	0.48	0.50	0.49	0.44	0.06
**8**	0.35	0.50	0.39	0.46	0.50	0.47	0.44	0.06

Motor Behavior: Grip Strength								
Time (weeks)	Animal Name						Average	SD
	CR1	CR2	CR3	CR4	CR5	CR6		
**Baseline**	723.33	794.50	730.67	748.83	842.43	765.83	767.60	44.73
**2**	580.95	468.52	674.08	652.62	490.65	603.48	578.38	83.75
**3**	566.77	645.53	820.63	713.52	781.28	586.40	685.69	103.67
**4**	855.03	708.90	844.95	723.32	766.82	537.25	739.38	116.03
**5**	755.73	758.15	752.50	714.72	701.70	678.38	726.86	33.47
**6**	720.30	823.47	739.35	659.18	667.08	609.70	703.18	74.90
**7**	789.45	803.98	716.23	805.50	729.87	650.82	749.31	61.53
**8**	729.32	846.30	670.15	682.32	790.72	797.40	752.70	70.03

Histology: Neuron Survival								
Distance from Implant (µm)	Animal Name						Average	SD
	CR1	CR2	CR3	CR4	CR5	CR6		
**0 - 50 µm**	46.13	2.56	56.23	68.63	36.77	47.29	42.94	22.51
**50 - 100 µm**	97.30	42.42	72.09	101.37	92.90	103.82	84.98	23.73
**100 - 150 µm**	90.28	82.25	86.03	97.74	103.72	108.44	94.74	10.29

Histology: Activiated Microglia/Macrophages								
Distance from Implant (µm)	Animal Name						Average	SD
	CR1	CR2	CR3	CR4	CR5	CR6		
**0 - 50 µm**	3.49	10.75	0.52	8.10	0.03	1.39	4.05	4.41
**50 - 100 µm**	0.09	0.73	0.28	0.56	0.05	0.22	0.32	0.27
**100 - 150 µm**	0.15	0.40	0.30	0.25	0.04	0.11	0.21	0.13

Histology: Astrocyte Reactivity								
Distance from Implant (µm)	Animal Name						Average	SD
	CR1	CR2	CR3	CR4	CR5	CR6		
**0 - 50 µm**	4.72	5.38	2.89	6.40	1.87	2.00	3.88	1.89
**50 - 100 µm**	1.73	2.84	1.13	1.83	0.39	0.60	1.42	0.90
**100 - 150 µm**	1.08	1.68	1.09	0.86	0.27	0.35	0.89	0.52

Histology: Blood Brain Barrier Permeability								
**Distance from Implant (µm)**	**Animal Name**						**Average**	**SD**
	**CR1**	**CR2**	**CR3**	**CR4**	**CR5**	**CR6**		
**0 - 50 µm**	63.14	18.63	17.45	25.35	2.59	23.61	25.13	20.28
**50 - 100 µm**	16.51	5.09	7.45	5.59	0.83	6.94	7.07	5.18
**100 - 150 µm**	4.39	1.74	3.33	2.32	0.68	2.23	2.45	1.28

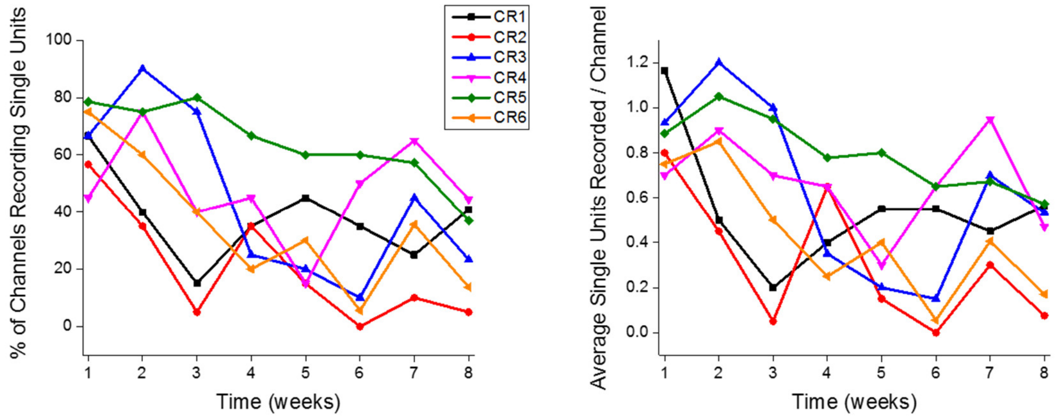

Figure SI1. Depiction of each animals' electrophysiological performance metrics individually plotted. The graph on the left demonstrates the percentage of working channels recording single unit activity and the graph on the right illustrates the average single units recorded per channel.

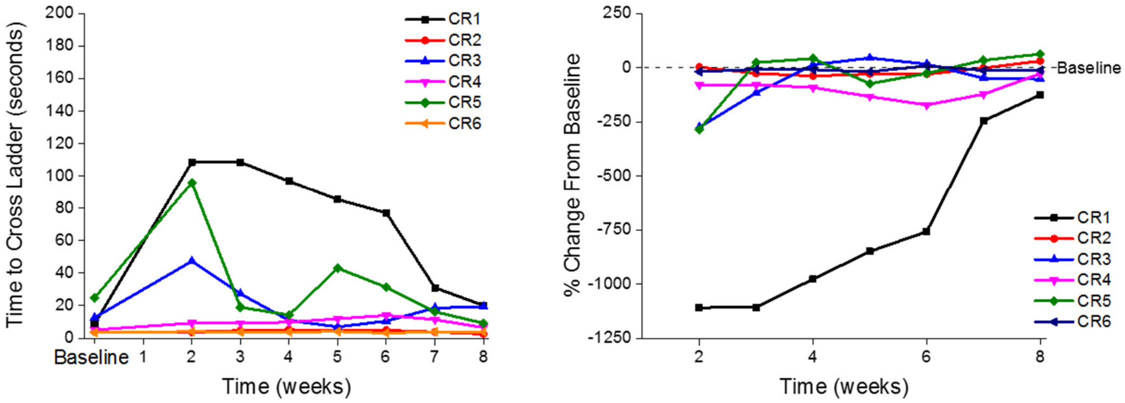

Figure SI2. Graphical representation of each animal's performance on the Ladder test. The graph on the left represents each animal's time to cross the ladder, while the graph on the right illustrates the normalized percent change in time to cross the ladder as compared to baseline.

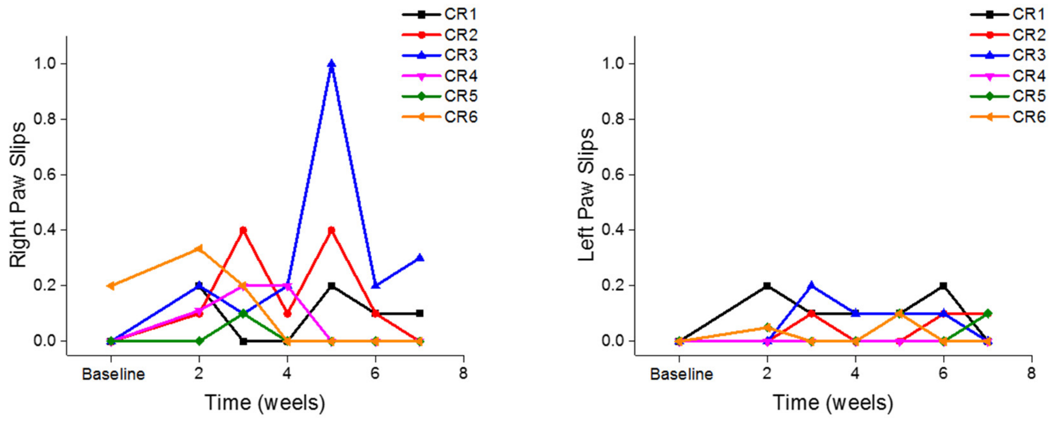

Figure SI3. Number of paw slip from each animal. The left graph represents the right paw slips and the right graph represents the left paw slips.

© 2020 by the authors. Licensee MDPI, Basel, Switzerland. This article is an open access article distributed under the terms and conditions of the Creative Commons Attribution (CC BY) license (http://creativecommons.org/licenses/by/4.0/).
